# A Key Factor for Psychosomatic Burden of Frontline Medical Staff: Occupational Pressure During the COVID-19 Pandemic in China

**DOI:** 10.3389/fpsyt.2020.590101

**Published:** 2021-01-18

**Authors:** Juanjuan Yi, Lijing Kang, Jun Li, Jianfang Gu

**Affiliations:** ^1^Department of Infectious Diseases and Public Health, Jockey Club College of Veterinary Medicine and Life Sciences, City University of Hong Kong, Hong Kong, China; ^2^State Key Laboratory of Medical Neurobiology, Department of Translational Neuroscience, Ministry of Education Frontiers Center for Brain Science, Institutes of Brain Science, Jing'an District Centre Hospital of Shanghai, Fudan University, Shanghai, China; ^3^School of Data Science, City University of Hong Kong, Hong Kong, China; ^4^Department of Health Service, The 980th Hospital of the Chinese People's Liberation Army Joint Logistics Support Force, Shijiazhuang, China; ^5^Wuhan Huoshenshan Hospital, Wuhan, China

**Keywords:** COVID-19, psychosomatic health, medical staff, risk factor, occupational health

## Abstract

The global outbreak of COVID-19 has severely affected the entire population, especially healthcare staff on the frontline, who bear heavy psychosomatic burdens. A cross-sectional study was conducted with 723 participants in China from April 26 to May 9, 2020. We evaluated the psychosomatic status, including depression, anxiety, quality of life, somatic symptoms, stress, sleep disturbances, and posttraumatic stress symptoms in different exposure groups. We explored the risk factors that affect psychosomatic burdens and analyzed the relationship between psychosomatic problems and medical occupations. We found that the psychosomatic burdens of medical staff were significantly greater than those of non-medical staff (*p* < 0.01) and were positively related with the number of COVID-19 patients they came in contact with. Occupational pressure was a key factor for healthcare staff's psychosomatic problems (*p* < 0.01 for quality of life, somatic symptoms, anxiety, depression, stress; *p* = 0.012 for sleep disturbances), and it had a strong canonical correlation (*p* < 0.01). Workload and time allocation (WTA), one of the subdimensional indicators of occupational pressure, was strongly correlated with psychosomatic indicators. We suggest that rationalization of WTA is a desirable approach for anti-epidemic medical employees to alleviate psychosomatic burdens. Public health interventions should be undertaken to reduce the occupational pressure on this special population, which is critical for mitigation. This study presents results regarding the psychosomatic burdens of the healthcare workforce related to occupational pressure and provides multilevel data with groups of different exposure risks for policymakers to protect medical personnel. These findings draw attention to the working environments of healthcare workers and provide applicable results for clinical practice.

## Introduction

With the outbreak of coronavirus disease 2019 (COVID-19) in December 2019, China first entered a state of disease resistance in Wuhan, Hubei Province (1). Currently, the epidemic has broken out in more than 210 countries or territories. Globally, as of November 20, 2020, there have been 56 million confirmed cases of COVID-19, including 1.3 million deaths reported to WHO, and the number of cases is still rising (2).

COVID-19 is highly contagious, and no effective drug is currently available. Frontline healthcare providers are facing huge dilemmas with uncontrollably rising numbers, a risk of personally being infected, a lack of medical resources, the suffering of patients, etc. Any of these difficulties can affect their physical and mental health. Numerous articles evaluating the mental health of the general population and healthcare workers have been published, generally focusing on two to three psychological evaluation indicators, such as anxiety and depression (3–9). Some reviews combined samples and mental indicators from different surveys for more general conclusions (10–12). However, there is a paucity of studies identifying the potential sources of psychological problems. There was substantial heterogeneity (*I*^2^ = 99.7%, *p* < 0.001) (11) in the combined analyses of different studies. Comprehensive psychological analysis focusing simultaneously on psychological and somatic symptoms is still lacking.

To identify the major source of the medical staff's psychosomatic problems in order to provide targeted mitigation measures, we systematically and completely compared the degree of seven psychosomatic problems in the different exposure groups, explored the risk factors for psychosomatic burdens, and analyzed the relationship between psychosomatic problems and medical occupation.

## Methods

### Study Design

An online questionnaire with the assistance of a questionnaire web platform (wenjuan.com) was completed by the participants (Supplementary Figure 1) from April 26 to May 9, 2020. The first part of the questionnaire included informed consent and demographic information, including age, sex, education, marital status, occupation, geographic location, mental problems before the outbreak, and working hours per day. Medical workers needed to answer additional questions including medical work experience, professional title, military personnel or not, department, antiepidemic experience, and hospital category. In the second part, we assessed psychosomatic problems during the peak period of COVID-19 in China using measurements of depression (Patient Health Questionnaire-9; PHQ-9 ≥5) (13), anxiety (Generalized Anxiety Disorder-7; GAD-7 ≥5) (13), quality of life (QOL; EuroQol visual analog scale; EQ-VAS) (14), somatic symptom load (Somatic Symptom Scale-8; SSS-8 ≥4) (15), stress (stress part of Depression Anxiety Stress Scales-21; DASS-stress ≥15) (16), sleep quality problems (Pittsburgh Sleep Quality Index; PSQI ≥5) (17), and posttraumatic stress symptoms (Posttraumatic Stress Symptoms Checklist-10; PTSS-10 ≥5) (18), while observing medical staff's occupational pressure (adapted from Nurse Job Stressor Questionnaire; NJSQ) (19). These are all proven psychometric instruments, and the scoring standards and grades were also consistent with the routine. In the third part of the questionnaire, we evaluated PTSD during the survey period when the outbreak was basically under control.

This study focused on the occupational pressure of healthcare staff during the epidemic. The NJSQ was produced by adapting the sources of stress inventory developed by H. Wheeler and R. Riding (20), and it is widely used in China (19, 21). It consists of five subscales: professional and career issues (PC; 7 items), workload and time allocation (WTA; 5 items), resource and environment problems (REP; 3 items), patient care and interactions (PCI; 11 items), and interpersonal relationships and management problems (IRMP; 9 items), totaling 35 items (Supplementary Table 1). In our survey, the PC part (e.g., “you had little opportunity to further study”) that medical staff would not encounter during the outbreak was excluded, and the word “nursing” was replaced with “healthcare service.” Cronbach's alpha and Kaiser–Meyer–Olkin (KMO) values were 0.941 and 0.909, respectively. Thus, all of the evaluation tools in this study have high reliability and validity (Supplementary Table 2).

Respondents answered the questionnaire anonymously and could choose to quit at any time during the process. Questionnaires with any unfinished questions were not recorded. The questionnaire could only be answered once from each WeChat account, computer, or mobile device to ensure that no one could fill it out repeatedly. The sample size estimation was based on the rule of thumb that logistic models should be used with a minimum of 10 outcome events per predictor variable (10 EPV rule) (22–24). As many samples as possible were collected during the survey period even when the 10 EPV rule were satisfied.

Online informed consent was obtained from participants. The study was approved by the ethics committee of the 980th Hospital of the Chinese PLA Joint Logistics Support Force.

### Data Collection

Nationwide participants were divided into medical staff (MS) and non-medical staff (NMS). According to the COVID-19 diagnosis and treatment plan formulated by the Ministry of Health, hospitals across the country were divided into different antiepidemic functions at the beginning of the outbreak by the health institutions in China. To fight against the pandemic, two specialized hospitals had been built in Wuhan to treat confirmed COVID-19 inpatients. Meanwhile, qualified hospitals had been designated as hospitals to treat fever patients, and the unselected hospitals (non-designated hospitals) did not accept fever patients. Therefore, the MS in different hospitals could be divided into three categories according to the number of COVID-19 patients they came into contact with: MS in the specialized hospitals on the frontline were the high-exposure group, MS in the designated hospitals were the low-exposure group, and MS in the non-designated hospitals were the non-exposure group.

To ensure collecting reliable data and valid response rate, the medical participants were mainly invited by researchers. Four types of data quality checks were conducted. First, questionnaires completed in <2 min were excluded from the analysis. Second, participants who had “severe” mental problems before the outbreak were excluded. Third, the questionnaire was set up with two repetitive questions. Participants who had different answers to the repetitive questions and the degree of difference was greater than two levels were excluded. Fourth, participants who were younger than 14 years old were excluded.

### Statistical Analysis

The data were analyzed using SPSS version 25 (IBM, Armonk, NY, USA) software. χ^2^ tests were used to compare group differences of categorical variables. Mann–Whitney tests or Kruskal–Wallis tests were used to compare two or more independent groups on continuous variables, which are non-normally distributed. Multivariate logistic regression analyses were used to select risk factors for psychosomatic problems. Canonical correlation analyses were used to explore the correlation between two sets of variables in the MS group. Significant difference was defined as two-tailed *p* < 0.05.

## Results

### Summary of the Study Population

A total of 742 respondents completed the questionnaire, and 19 were excluded after quality control. The sample of this study was from more than 19 provinces in China. Four provinces with sample sizes >50 each were Hubei, Shānxi, Hebei, and Shanghai (Supplementary Table 3). Of the 723 participants, the majority were female (59.5%), married (66.9%), had a bachelor's degree (46.9%), lived outside Hubei (73.2%), had no previous mental problems (97.5%), working hours per day <4 (38.3%), and their mean age was 34.71 years (Supplementary Table 4).

### Psychosomatic Problems in Different Exposure Groups

There was no significant difference in mental problems before the COVID-19 outbreak between the MS and NMS groups (*p* > 0.05) based on the questionnaire (Supplementary Table 5). Table 1 shows that somatic symptoms, anxiety, depression, stress, and sleep disorders had higher scores, and QOL had lower scores in MS than NMS (*p* < 0.01) during the epidemic.

**Table 1:** Comparison of psychosomatic problems between medical staff (MS) and non-medical staff (NMS).

**Variables**	**NMS**	**MS**	**Total**
	**(*n* = 552)**	**(*n* = 171)**	**(*n* = 723)**
QOL	79.41 ± 24.18	75.57 ± 22.51**	78.5 ± 23.84
Somatic Symptom	1.73 ± 2.70	4.14 ± 4.45**	2.30 ± 3.36
Anxiety	3.77 ± 3.70	5.65 ± 4.31**	4.21 ± 3.93
Depression	3.34 ± 4.09	4.63 ± 4.27**	3.64 ± 4.17
Stress	5.50 ± 7.33	7.85 ± 7.52**	6.06 ± 7.44
Sleep	4.26 ± 3.54	6.73 ± 4.29**	4.84 ± 3.87
PTSS	1.47 ± 2.21	1.47 ± 2.26	1.47 ± 2.22

Compared with NMS, **p < 0.01.

Furthermore, we analyzed the psychosomatic problems of the different categories of the MS. The results showed that the scoring trend was increasing in the assessment of somatic symptoms, anxiety, depression, stress, sleep quality problems, and occupational pressure, and was declining in QOL from the non-exposure group to the high-exposure group (Table 2). When compared with the high-exposure group, the non-exposure group showed significant differences in all of the variables above (*p* < 0.01), and the low-exposure group had significant differences in somatic symptoms (*p* < 0.01), anxiety (*p* < 0.05), stress (*p* < 0.01), sleep (*p* < 0.01), occupational pressure (*p* < 0.05), and QOL (*p* < 0.01). The somatic symptoms (*p* < 0.01) and occupational pressure (*p* < 0.05) scores of low-exposure group were significantly higher than those of the non-exposure group. Statistical differences in PTSS were not found among any of the groups.

**Table 2:** Comparison of psychosomatic indicators and occupational pressure between different exposure groups in medical staff (MS).

**Variables**	**High-exposure group (*n* = 72)**	**Low-exposure group (*n* = 51)**	**Non-exposure group (*n* = 48)**
QOL	70.85 ± 21.22	78.90 ± 21.82**	79.13 ± 24.24**
Somatic symptom	6.32 ± 4.65	3.51 ± 4.24**	1.54 ± 2.31**^##^
Anxiety	7.08 ± 4.23	5.31 ± 4.18*	3.85 ± 3.89**
Depression	5.60 ± 4.21	4.37 ± 4.28	3.44 ± 4.09**
Stress	10.08 ± 7.14	7.02 ± 7.24**	5.38 ± 7.56**
Sleep	8.88 ± 3.94	5.27 ± 3.57**	5.04 ± 4.15**
PTSS	1.22 ± 2.04	1.57 ± 2.54	1.75 ± 2.26
Occupational pressure	8.06 ± 1.91	7.16 ± 2.56*	6.05 ± 2.05**^#^

Compared with high-exposure group, *p < 0.05, **p < 0.01.

*Compared with low-exposure group, ^#^p < 0.05, ^##^p < 0.01*.

### Risk Factors for Psychosomatic Manifestations

To select independent risk factors from among all of the characteristic variables mentioned in the methods, multiple logistic regression analyses (Table 3) were performed. The results showed that occupational pressure was a risk factor for the decline in QOL in the medical group and was inversely related to the QOL scores [*p* < 0.01; odds ratio (OR) = 0.19; 95% CI, 0.07–0.49]. For MS's somatic symptoms, education (*p* = 0.02; OR = 1.77; 95% CI, 1.1–2.85), and occupational pressure (*p* < 0.01; OR = 8.08; 95% CI, 2.96–22.02) were risk factors, while living outside Hubei (*p* < 0.01; OR = 0.33; 95% CI, 0.16–0.66) was a protective factor. Being female (*p* = 0.028; OR = 2.31; 95% CI, 1.09–4.88) and occupational pressure (*p* < 0.01; OR = 10.94; 95% CI, 3.88–30.74) were risk factors for anxiety in MS, and education (*p* < 0.01; OR = 1.27; 95% CI, 1.08–1.5), location (*p* < 0.01; OR = 0.56; 95% CI, 0.4–0.78), and daily working hours (p < 0.01; OR = 1.31; 95% CI, 1.07–1.6) were factors related to anxiety in NMS. In the depression model, lack of prior antiepidemic experience (*p* = 0.011; OR = 2.14; 95% CI, 1.19–3.85) and occupational pressure (*p* < 0.01; OR = 12.43; 95% CI, 4.32–35.8) were risk factors, and living outside Hubei (*p* = 0.013; OR = 0.43; 95% CI, 0.22–0.83) was a protective factor among MS. Daily working hours (*p* = 0.023; OR = 1.28; 95% CI, 1.03–1.57) were a risk factor for depression in NMS. The stress of MS came from daily working hours (*p* = 0.033; OR = 1.65; 95% CI, 1.04–2.62) and occupational pressure (*p* < 0.01; OR = 6.67; 95% CI, 2.31–19.24), while for NMS, the stress came from sex (*p* = 0.036; OR = 1.99; 95% CI, 1.05–3.79). Three independent variables were influencing factors for MS's sleep disturbances: education (*p* < 0.01; OR = 2.29; 95% CI, 1.46–3.61), location (*p* < 0.01; OR = 0.21; 95% CI, 0.11–0.41), and occupational pressure (*p* = 0.012; OR = 3.54; 95% CI, 1.32–9.49).

**Table 3:** Outcomes of psychosomatic problems.

**Variables**	**NMS**		**MS**	
	***p-*value**	**OR(95% CI)**	***p-*value**	**OR(95% CI)**
*Models for QOL*	No variables were entered			
Occupational pressure	–		<0.01	0.19(0.07, 0.49)
*Models for Somatic Symptom*	No variables were entered			
Education	–		0.02	1.77(1.1, 2.85)
Location			<0.01	0.33(0.16, 0.66)
Occupational pressure			<0.01	8.08(2.96, 22.02)
*Models for Anxiety*				
Education	<0.01	1.27(1.08, 1.5)	–	
Location	<0.01	0.56(0.4, 0.78)		
Working hours per day	<0.01	1.31(1.07, 1.6)		
Sex	–		0.028	2.31(1.09, 4.88)
Occupational pressure			<0.01	10.94(3.88, 30.78)
*Models for Depression*				
Working hours per day	0.023	1.28(1.03, 1.57)		
Anti-epidemic experience	–		0.011	2.14(1.19, 3.85)
Location			0.013	0.43(0.22, 0.83)
Occupational pressure			<0.01	12.43(4.32, 35.8)
*Models for Stress*				
Working hours per day	–		0.033	1.65(1.04, 2.62)
Sex	0.035	1.99(1.05, 3.80)	–	
Occupational pressure	–		<0.01	6.67(2.31, 19.24)
*Models for Sleep Quality*	No variables were entered			
Education	–		<0.01	2.29(1.46, 3.61)
Location			<0.01	0.21(0.11, 0.41)
Occupational pressure			0.012	3.54(1.32, 9.49)
*Models for PTSS*	No variables were entered			

### Relationships Between Occupational Indicators and Psychosomatic Indicators of MS

**Figure 1:**
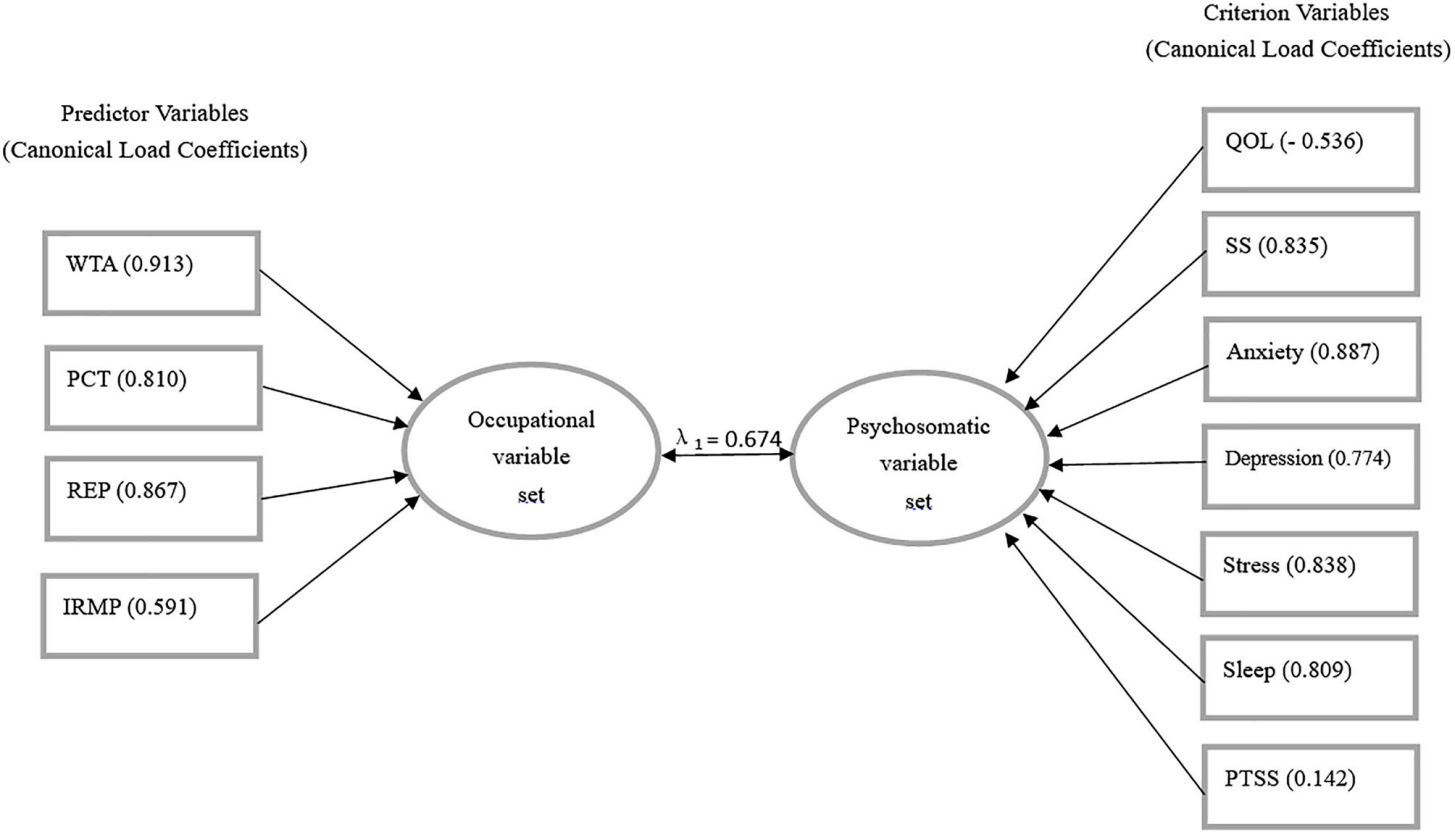
The first pair of canonical correlation variables.

Canonical correlation analyses (Figure 1) were used to explore the correlations between the occupational indicators (WTA, REP, PCI, and IRMP) and the psychosomatic indicators. The correlation between the first pair of canonical variate groups was maximized (correlation coefficient λ_1_ = 0.674, Wilks' lambda = 0.395, *F* = 6.190, *p* < 0.01). The origin variable that has a large absolute value of canonical load (CL > 0.5) means it has a large role in the variable set, and the greater the value, the more its contributions will be. The sign of the variable coefficient determines the direction of the relationship.

The canonical load of the variables indicated that the sequence of contributions to the synthetic variate of the occupational pressure was WTA, REP, PCI, and IRMP (with CL = 0.913, 0.867, 0.810, and 0.591). Besides, the canonical load of anxiety, stress, somatic symptoms (SS), sleep disturbances, depression, and QOL showed that they were the primary contributors (with CL = 0.887, 0.838, 0.835, 0.809, 0.774, and 0.556) to the synthetic variate of psychosomatic burdens. All occupational indicators were positively correlated with other psychosomatic indicators except a negative correlation with QOL.

## Discussion

COVID-19 has resulted in an unprecedented international public health response and attracted attention around the world. Compared to the general population, healthcare workers are being confronted with dire challenges. Recent studies suggest that the pandemic has caused a high prevalence of anxiety and depression among the adult population, especially among medical workers (3–12). Additionally, some studies have explored the risk factors (e.g., sex, region) of different populations in addition to performing prevalence evaluations (25–28). However, the source of psychological problems and the impact of medical occupation on psychological indicators during the pandemic are not scientifically understood.

Our data showed that the mean QOL scores of the frontline MS and NMS were 70.85 and 79.41, respectively, during the outbreak of COVID-19, both lower than the score of the general population (85.4) (14) before the epidemic. Interestingly, the more COVID-19 patients the MS were exposed to, the higher their scores of somatic symptoms, anxiety, depression, stress, and sleep disorders, and the frontline MS had the highest scores. Compared to the NMS, the stress score nearly doubled in the non-exposure MS, while there was no significant difference for it or for other indicators (Supplementary Table 6). Such insignificantly different levels of psychosomatic problems between NMS and non-exposure MS indicate that the occupational difference itself may not result in psychosomatic differences. Future studies with a larger sample size are needed to validate this discovery. In our study, a significant difference in PTSD related to COVID-19 between MS and NMS was not found. However, PTSD should not be ignored, as the proportion of MS with PTSD was 13.5%. A systematic review reported that the prevalence of PTSD ranged from 3% (2–4%) to 16% (15–17%) among healthcare workers (11), similar to the results of our study. A previous study showed that approximately 10% of hospital employees had SARS-related PTSD in Beijing during the 3 year period following the outbreak (29). The prevalence of PTSD varies in different studies and may be related to regions, populations, duration of the pandemic, etc.

Occupational pressure was the critical risk factor for all statistically significant psychosomatic indicators of MS during the epidemic. Longer working hours per day resulted in a longer exposure to public environments and a higher infection risk, which contributed to NMS's anxiety and depression. Location was a risk factor because Wuhan and other cities in Hubei were the hardest-hit areas. People who are closer to the epidemic center are more likely to bear psychological pressure. Education was a risk factor for somatic symptoms and sleep quality among MS and anxiety among NMS. People with a higher education are more aware of the characteristics (completely unknown, highly contagious, and no available drugs) of COVID-19. Women were more prone to anxiety and stress, which is consistent with a previous research (30). When we carried out an in-depth exploration of the risk factors in the three exposure subgroups of the medical staff, we found that prior antiepidemic experience was also very important for frontline medical staff (*p* = 0.046 for QOL; *p* = 0.19 for somatic symptoms; *p* < 0.01 for depression, Supplementary Table 7). That is, the medical staff who have experienced the outbreak of other epidemics were able to deal with the psychosomatic problems better in the harsh environment of frontline health care.

Finally, the results of the canonical correlation analyses validated the evidence of the psychosomatic harms of exposure to occupational pressure. This study also revealed the key variables of the subdimensions of occupational pressure in the relationship between occupational pressure and psychosomatic well-being. The analytical results showed that the variables of WTA and REP ranked in the top 2 in influencing psychosomatic burdens. However, previous studies usually did not consider these relationships (3–12, 25–28, 31). Our study presented the correlations between four subdimensions of occupational pressure and the degree of seven psychosomatic burdens, which prompted us to seek reliable solutions from WTA and REP: (a) to reduce the workload, (b) to increase the number of frontline medical staff, (c) to give sufficient time for medical work and to reduce other non-medical work, (d) to improve the working environment, (e) to increase the supply of medical equipment, and (f) to reduce congestion in the wards. WTA, REP, and PCI in the high-exposure group were significantly higher than those in the non-exposure group. These subdimensional differences in occupational pressure indicators should be given more attention among frontline medical staff, and the higher WTA in the low-exposure group should not be ignored (Supplementary Table 8).

This study divided medical staff into subgroups according to their exposure risk, which is particularly important for the hardest hit countries since the workload of medical staff soars due to the pandemic. Recent meta-analyses found that the prevalence of anxiety and depression was similar between healthcare workers and the general public (11, 28), while other studies revealed that healthcare workers had a higher prevalence of anxiety and depression (9, 31). The contradictions among these studies may be caused by sampling bias or a failure to properly distinguish exposure groups. The significant difference in psychosomatic indicators between the MS and NMS groups and the insignificant difference in these indicators between the non-exposure MS and NMS groups in our study could reconcile the controversy in previous studies. However, several limitations of this study merit discussion. First, selection bias could exist due to the use of an online survey. Although we carried out very strict *post hoc* quality control in the investigation process, potential sample bias could still exist. Second, the long-term mental health implications can hardly be inferred from our cross-sectional study. Future longitudinal studies would be designed prospectively with follow-up observations of psychological status over time.

In summary, antiepidemic MS all bear heavy psychosomatic burdens in different countries during the COVID-19 epidemic. Our findings demonstrate that the psychosomatic burdens of MS are more serious than those of NMS and increase with the number of COVID-19 patients they take care of. We emphasize that supervisors should not ignore these people's somatic symptoms, anxiety, depression, stress, sleep disorders, and PTSD, especially among the frontline healthcare workers.

Importantly, we also showed that among all risk factors, occupational pressure is a key factor for healthcare staff's psychosomatic problems during the pandemic. Reducing occupational pressure is critical for relief. The variables WTA and REP play the main roles in influencing psychosomatic burdens. Seeking reliable solutions from the findings will be useful to guide public health and professional environment response measures worldwide. It is expected that policymakers will pay attention and provide recovery programs to the MS, especially in this difficult period.

## Data Availability Statement

The raw data supporting the conclusions of this article will be made available by the authors, without undue reservation.

## Ethics Statement

The studies involving human participants were reviewed and approved by The ethics committee of the 980th Hospital of the Chinese PLA Joint Logistics Support Force. Written informed consent for participation was not required for this study in accordance with the national legislation and the institutional requirements.

## Author Contributions

JY and LK conceived the research, designed the questionnaire, and wrote the manuscript. JY, LK, and JG promoted the collection of data to ensure the reliability of the data. JY, LK, and JL conducted data analysis. JG and JL supervised the project and revised the manuscript. All authors contributed to the article and approved the submitted version.

## Conflict of Interest

The authors declare that the research was conducted in the absence of any commercial or financial relationships that could be construed as a potential conflict of interest. The handling Editor declared a shared affiliation, though no other collaboration, with several of the authors JY, JL.
