# Supplementary material for: A Key Factor for Psychosomatic Burden of Frontline Medical Staff: Occupational Pressure During the COVID-19 Pandemic in China
Source: Front Psychiatry. 2021 Jan 18;11:590101. doi: 10.3389/fpsyt.2020.590101 (PMC7848019; doi:10.3389/fpsyt.2020.590101)

Supplementary Table 1. NJSQ

		none	mild	moderate	much	extreme
1	The social status of nursing work is too low					
2	Little opportunity to further study					
3	Low wages and other benefits					
4	Too few opportunities for promotion					
5	Often shifting work					
6	Less independence at work					
7	Unclear division of work					
8	Workload is too heavy					
9	Number of working nurses is not enough					
10	No time for psychological care of patients					
11	Too much non-nursing work					
12	Too much useless paperwork					
13	Poor working environment					
14	Insufficient instruments and equipment required					
15	Crowded Ward					
16	Worried about errors at work					
17	The work is not understood by patients and family members					
18	Patient's condition is too severe					

19	Patient's family number is impolite					
20	Patient's request is too high or too much					
21	Patient is impolite					
22	Patient does not cooperate					
23	Knowledge learned cannot meet the psychological needs of patients and their families					
24	Lack of knowledge about patient education					
25	Worried that nursing operations will cause pain to patients					
26	Sudden death of nursing patient					
27	Lack of understanding and respect from other health workers					
28	Insufficient understanding and support of managers					
29	Too much criticism from managers					
30	Colleagues are too picky about nursing work					
31	Lack of understanding and support among colleagues					
32	Conflict with the managers					
33	Difficult to work with some colleagues in the ward					

34	Conflict with the colleagues					
35	Lack of friendly and cooperative atmosphere among colleagues					

Supplementary Table 2. Reliability and validity of assessment tools

Scale	Cronbach α	KMO	Bartlett test *p* value
SSS-8	0.845	0.873	< 0.001
GAD-7	0.926	0.923	< 0.001
PHQ-9	0.919	0.931	< 0.001
DASS-stress	0,930	0.921	< 0.001
PSQI	0.905	0.872	< 0.001
PTSS-10	0.839	0.890	< 0.001
NJSQ	0.941	0.909	< 0.001

Cronbach α> 0.8, the reliability is high; KMO> 0.8, the validity is high; p< 0.05, Factor analysis can be performed.

Supplementary Table 3. Sample distribution in different regions of China

Province	Number	Province	Number	Province	Number
Hubei	194	Hunan	16	Jiangxi	4
Shānxi	192	Shandong	13	Tianjin	2
Hebei	115	Heilongjiang	12	Shănxi	2
Shanghai	69	Henan	9	Gansu	2
Anhui	22	Guangdong	6	Hainan	1
Beijing	21	Neimenggu	6	International cargo ship	1
Jiangsu	18	Liaoning	4	Others (not specified)	14

Supplementary Table 4. Demographic characteristics of study population

Characteristics	NMS<br>(n= 552)	MS<br>(n= 171)	Total<br>(n= 723)
Sex, n(%)			
Female	315(57.1)	115(67.3)	430(59.5)
Male	237(42.9)	56(32.7)	293(40.5)
Age, *Mean* ± *SD*	34.32±10.39	35.99±8.01	34.71±9.90
Marital status, n(%)			
Single	181(32.8)	36(21.1)	217(30.0)
Married	353(63.9)	131(76.6)	484(66.9)
Others	18(3.3)	4(2.3)	22(3.0)
Education, n(%)			
Junior high school or below	33(6.0)	0(0.0)	33(4.6)
Vocational high school education	108(19.6)	9(5.3)	117(16.2)
College	102(18.5)	18(10.5)	120(16.6)
Bachelor	235(42.6)	104(60.8)	339(46.9)
Master	62(11.2)	30(17.5)	92(12.7)
PhD	12(2.2)	10(5.8)	22(3.0)
Geographic Location, n(%)			
Wuhan	22(4.0)	84(49.1)	106(14.7)
Other parts of Hubei	78(14.1)	10(5.8)	88(12.2)
Outside Hubei	452(81.9)	77(45.0)	529(73.2)
Occupation, n(%)			
MS		171(100.0)	171(23.7)
Farmer	21(3.8)		21(3.0)
Workman	44(7.9)		44(6.1)

Businessman	16(2.8)		16(2.2)
Service staff	35(6.3)		35(4.8)
Public functionary	150(27.1)		150(20.7)
Intellectual	66(11.9)		66(9.1)
Enterprise staff	108(19.5)		108(14.9)
Other	112(20.2)		112(15.5)
Working hours per day, n(%)			
<4 hours	264(47.8)	13(7.6)	277(38.3)
4-8 hours	174(31.5)	97(56.7)	271(37.5)
8-12 hours	96(17.4)	47(27.5)	143(19.8)
＞12 hours	18(3.3)	14(8.2)	32(4.4)
Medical work experience, n(%)			
<3 years		22(12.9)	
3-5 years		17(9.9)	
6-10 years	-	34(19.9)	-
＞10 years		98(57.3)	
Department, n(%)			
Clinic		57(33.3)	
Nursing		63(36.8)	
Administration	-	6(3.5)	-
Others		45(26.3)	
Professional title, n(%)			
None		13(7.6)	
Junior		55(32.2)	
Intermediate	-	88(51.5)	-
Associate senior		12(7.0)	

Senior		3(1.8)	
Military medical personnel, n(%)			
Yes	-	91(53.2)	-
No		80(46.8)	
Anti-epidemic experience, n(%)			
Yes		20(11.7)	
No, but trained	-	82(48.0)	-
None		69(40.4)	
Occupational pressure, *Mean* ± *SD*	-	7.23±2.30	-

Supplementary Table 5. Mental problems before the epidemic

Status	NMS (n= 552)	MS (n= 171)	χ^2	*p value*
Mental problems before epidemic, n(%)			1.290	0.525
None	537(97.3)	168(98.2)		
Mild	11(2.0)	3(1.8)		
Medium	4(0.7)	0(0.0)		

Supplementary Table 6. Comparison of NMS and non-exposure MS

Variables	NMS (n = 552)	MS (Non-exposure group) (n =48)
Quality of Life	79.41±24.18	79.13±24.24
Somatic Symptom	1.73±2.70	1.54±2.31
Anxiety	3.77±3.70	3.85±3.89
Depression	3.34±4.09	3.44±4.09
Stress	2.75±3.67	5.38±7.56

Sleep Quality	4.26±3.54	5.04±4.15
PTSS	1.47±2.21	1.75±2.26

Supplementary Table 7. Exploratory analyses of risk factors in different exposure group

Variables	High-exposure group (n= 72)		Low-exposure group (n= 51)		Non-exposure group (n= 48)	
	p value	*OR(95% CI)*	*p value*	*OR(95% CI)*	*p value*	*OR(95% CI)*
Models for quality of life			No variables were entered		No variables were entered	
Anti-epidemic experience	0.046	0.44(0.19, 0.98)				
Occupational pressure	<0.01	0.07(0.02, 0.34)				
Models for Somatic Symptom						
Education			0.014	3.35(1.28, 8.81)		
Location			0.025	0.23(0.06, 0.83)	0.035	<0.001(0, 0.67)
Anti-epidemic experience	0.019	2.53(1.17, 5.49)				
Occupational pressure	<0.01	16.83(3.84, 73.85)				
Models for Anxiety						
Sex	0.029	3.47(1.14, 10.57)				
Medical work experience			<0.01	5.4(1.57, 18.54)		
Education			0.01	3.16(1.32, 7.55)		
Location					<0.01	0.03(0, 0.26)
Occupational pressure	<0.01	28.05(5.43, 144.89)				
Models for Depression						
Anti-epidemic experience	<0.01	3.35(1.42, 7.89)				
Education			0.049	2.6(1, 6.76)		
Location					<0.01	0.03(0, 0.28)
Occupational pressure	<0.01	39.69(7.22, 218.11)				
Models for Stress						
Working hours per day					0.023	3.28(1.18, 9.18)
Education			0.013	3.12(1.27, 7.69)		

Medical work experience			0.045	3.5(1.03, 11.94)		
Occupational pressure	<0.01	33.25(5.44, 203.36)				
Models for Sleep Quality						
Education			<0.01	6.88(2.26, 20.88)		
Location			0.01	0.13(0.03, 0.61)	<0.01	0.05(0.01, 0.47)
Professional title			0.015	0.3(0.11, 0.79)		
Military personnel or no			<0.01	0.04(0, 0.33)		
Occupational pressure	<0.01	8.51(1.97, 36.82)				
Working hours per day					0.044	2.42(1.03, 5.72)
Models for PTSS	No variables were entered					
Location					0.02	0.005(0, 0.432)
Medical work experience			0.049	33.09(1.02, 1071.71)		

Note: The results maybe not robust enough due to the limitation of sample size in each regression model.

Supplementary Table 8. Comparison of occupational indicators between different exposure group in MS

Indicators	High-exposure group (n= 72)	Low-exposure group (n= 51)	Non-exposure group (n= 48)
WTA	2.83±0.74	2.19±0.90**	1.68±0.68**##
REP	2.13±0.74	1.82±0.71*	1.57±0.68**
PCI	1.79±0.48	1.68±0.67	1.51±0.55*
IRMP	1.31±0.40	1.47±0.72	1.29±0.53

Compared with high-exposure group, *$p < 0.05$, **$p < 0.01$. Compared with Low-exposure group, #$p < 0.05$, ##$p < 0.01$.

Supplementary Table 9. PTSS-10

Below you find ten questions about common reactions among people who have been involved in an accident or a disaster. Kindly answer each question to indicate how you have been reacting during the last seven days. If in doubt, take the alternative closest to how you feel or react.

Please answer all the questions.

During the last seven days you have had:	
1.	Difficulty with sleep
2.	Nightmares about the accident or disaster
3.	Depressed feelings
4.	Tendencies to jump or startle at sudden noises or unexpected movements
5.	Tendencies to withdraw myself from others
6.	Irritable feelings (I am easily getting irritable or infuriated)
7.	Frequent swings in mood
8.	Bad conscience, self accusations or guilt

9.	Fears when approaching the place of the accident or situations that remind me of it
10.	Tensions in my body

Supplementary Figure 1. Sampling process

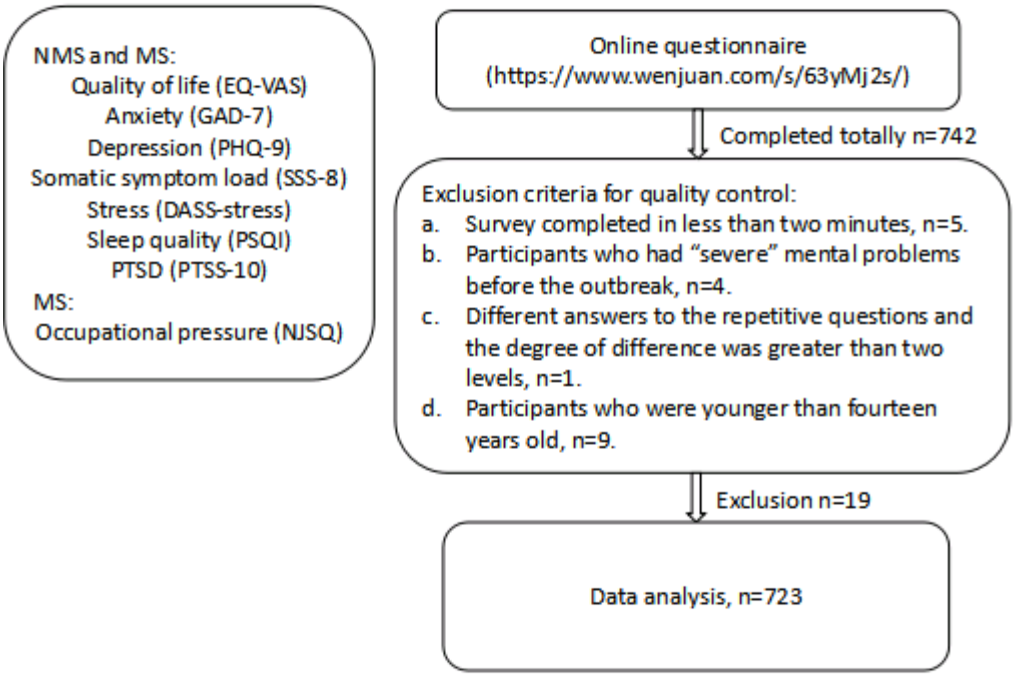

Supplement: Supplementary file 1 [file Data_Sheet_1.pdf]
